# Induction of apoptosis and proliferation inhibition of hepatocellular carcinoma by 6-chloro-2-methoxy-*N*-(phenylmethyl)-9-acridinamine (BA): in vitro and vivo studies

**DOI:** 10.1186/s12935-017-0435-5

**Published:** 2017-07-03

**Authors:** Yun Huang, Guohua Liu, Feng Yang, Xiaowei Xing, Ying Li, Zhijun Huang, Hong Yuan

**Affiliations:** 1 Center of Clinical Pharmacology, The Third Xiang Ya Hospital, Central South University, Changsha, 410013 China; 2 Department of Pharmacy, Ningbo City Medical Treatment Center Lihuili Hospital, Ningbo, 315000 China; 3 Department of Pharmacy, The First Affiliated Hospital of Shantou University Medical College, Shantou, 515041 China; 4 National & Local Joint Engineering Laboratory for the Technology of Clinical Drug Evaluation, Changsha, 410013 China

**Keywords:** BA, Sponge, Hepatocellular carcinoma, PI3K/Akt

## Abstract

**Background:**

6-Chloro-2-methoxy-*N*-(phenylmethyl)-9-acridinamine (BA), a novel sponge-derived compound, has been reported to elicit a cytotoxic effect by inhibiting cell proliferation.

**Methods:**

In this study, we investigated the anti-tumor effect of BA in human hepatocellular carcinoma (HCC) in vitro and in vivo using SMMC-7721 cells. The impact of BA on SMMC-7721 cells was determined by proliferation (clonogenicity and MTT), apoptosis (flow cytometry with annexin V-FITC labeling) and tumor cell migration (Transwell). Apoptosis-related molecules in the PI3K/AKT signaling pathway were examined via Western blotting. We also evaluated the effects of BA on tumor growth using a xenograft nude mouse model.

**Results:**

The data showed that BA induced dose-dependent cytotoxicity, anti-proliferation, anti-migration and apoptosis in SMMC-7721 cells, accompanied by activation of caspase-3 and a decreased level of caspase-9. Moreover, BA decreased PI3K and p-AKT levels, which indicated the cytotoxicity of BA through the PI3K/Akt pathway. Finally, we confirmed that BA inhibited tumor growth in an HCC xenograft mouse model.

**Conclusions:**

We concluded that BA induced apoptosis and decreased PI3K and p-AKT expression in human HCC with no effect on the liver, kidney, spleen or lungs. These findings suggest that BA could provide a novel strategy for the treatment of HCC.

**Electronic supplementary material:**

The online version of this article (doi:10.1186/s12935-017-0435-5) contains supplementary material, which is available to authorized users.

## Background

Primary liver cancer or hepatocellular carcinoma (HCC) is one of the most common malignancies worldwide. The Global Burden of Disease Study ranks HCC as the twenty-ninth most common cause of death globally, the sixth most common cancer and the third most lethal in 2013 [1], with an estimated 818,000 deaths worldwide [2]. HCC represents a major health challenge with a significant and ever-increasing global impact. As a result, therapeutic strategies targeting HCC have significantly advanced in recent years, among them surgical resection, liver transplantation, radiofrequency ablation (RFA) and transarterial chemoembolization [3]. Nevertheless, the long-term prognosis for HCC remains poor due to its high recurrence and metastasis [4]. Currently, surgery resection is the best treatment for HCC. However, most patients are ineligible for surgery because of their health condition or late diagnosis. Therefore, certain chemotherapeutic therapies have become the most dependable option as a palliative treatment to prolong life, often with poor quality.

Chemotherapeutic drugs targeting HCC such as doxorubicin, gemcitabine, oxaliplatin, cisplatin and 5-fluorouracil are a common treatment for systemic therapy. However, these treatments have not demonstrated satisfactory results. For example, doxorubicin has been routinely used as the sole drug for unresectable HCC, with a response rate of 20% and a median survival time of 4 months [5]. Other drugs either used alone or in combination have been reported not to provide satisfactory survival benefits [6]. The development of the targeted drug sorafenib, a multi-tyrosine kinase inhibitor, has improved survival rates and has been recommended to treat advanced stage (C) HCC [7]. However, sorafenib only improved life expectancy by 3 months compared to placebo [6]. Therefore, it is urgent to find new targets and possible molecular pathways for HCC treatment.

Natural sources including plants have a long history of medicinal use across many cultures. More than 3000 plant species have reportedly been used in anti-cancer treatment. Besides being used in medicine, natural products also work as lead compounds in generating new drugs via combinatorial biosynthesis or chemical synthesis [8, 9]. As the largest natural resource, marine natural products have attracted increasing attention for use in new anticancer drug development in recent years due to the chemical and biological diversity of the marine environment. Sponges, a sessile organism heavily involved in maintaining the marine ecosystem, exhibit significant antitumor, anti-inflammatory, and anti-viral functions. A variety of chemical compounds extracted and isolated from sponges, including macrocyclic polyether, lactone, diterpene, polyketide and alkaloid, have been used to treat cancer analgesia, allergy, and cognitive diseases [10, 11].

6-Chloro-2-methoxy-*N*-(phenylmethyl)-9-acridinamine (BA), a novel sponge derived compound, has been reported to elicit a cytotoxic effect inhibit cell proliferation [12]. In this study, we further investigated BA-induced inhibition of SMMC-7721 cell growth by analyzing its effect on apoptosis and cell migration, and we also determined the involvement of the PI3K/AKT pathway in BA mediated cell growth inhibition. Furthermore, we used a xenograft mouse model to confirm our findings in vivo.

## Methods

### Structural identification of BA

BA was extracted from sponges *(species)*, and its structure was optimized by the Graduate School of Shenzhen, Tsinghua University (purity >98%, HPLC) [13]. The Total mass of BA was identified by mass spectrometry (MS). Structural identification of BA was done by H-nuclear magnetic resonance spectroscopy (NMR) and C-NMR (as shown in Additional file 1: M1, Additional file 2: M2, Additional file 3: M3).

### Assessment of BA’s anti-hepatocellular carcinoma effect in vitro

#### Cell culture

The human hepatocarcinoma cell line SMMC-7721 and normal hepatocyte cell line LO2 were purchased from the Central Laboratory of Xiangya, Center South University. Both cell lines were cultured in DMEM media (Hyclone, USA), supplemented with 10% fetal bovine serum (Sijiqing, Zhejiang, China) in a humidified atmosphere containing 5% CO2 at 37 °C. The cells were dissociated using 0.25% trypsin and 0.02% EDTA solution and resuspended into fresh medium once every 2–3 days.

#### Measurement of cell viability

Cell viability was measured by 3-(4,5-dimethylthiazol-2-yl)-2,5-diphenyl tetrazolium (MTT, Sigma-Aldrich). Cells were seeded in a 96-well microplate (4500 cells/well) containing the relevant cell culture medium and incubated at 37 °C overnight before BA treatments. After being treated for the indicated times, the cells were incubated with a medium containing MTT for 4–6 h at 37 °C. Then the absorbance was detected on a BioTek Elx 800 ELISA reader (Winooski, VT, USA) at a wavelength of 490 nm.

### Clonogenic survival assay

The long-term clonogenic survival assay was used here based on the methods previously described [14]. Cells were seeded into 6-well tissue culture plates containing a complete medium at a density of 1.5 × 10^4^ cells per well. After attachment, cells were exposed to various concentrations of BA for 24 h, using 0.1% DMSO as a negative control. The cells were then cultured with a 10% FBS-DMEM medium until colonies were visible. The colonies were then fixed in 4% (w/v) glutaraldehyde, and stained with 0.1% (w/v) crystal violet. Colony formation rate = (number of clones/seeded cells) × 100%.

### Apoptosis assays

SMMC-7721 cells were seeded in 6 well culture plates containing a cell culture medium and were then treated with various concentrations of BA (2, 4, 8 and 16 μg/ml) for 24 h, using BA (0 μg/ml) as blank control. After being stained with hematoxylin and eosin (H&E), the morphological variations were observed and photographed with a camera attached to the microscope (Olympus, Japan) .

The apoptotic cells were quantified (percentage) using an Annexin V-fluorescein isothiocyanate (FITC)/propidium iodide (PI) apoptosis detection kit (BD). Cells were seeded at a density of 2 × 10^5^ cells per well in 6-well plates. After treatment, cells were harvested and counted using the TC10 Cell Counter (Bio-Rad, USA) and Annexin-V-FITC/PI labeling was performed according to the manufacturer’s instructions (Beyotime, China). Approximate fluorescence excitation maxima: 488 and 540 in nm. The stained cells were analyzed with a flow cytometer.

Cell cycle status was determined by measuring cellular DNA content after staining with PI. Briefly, cells were harvested and washed in PBS, then fixed in cold 70% ethanol. And drop wise was added to the pellet while vortexing, which should ensure fixation of all cells and minimize clumping. Then cells were fixed for another 30 min at 4 °C. After washed 2× in PBS, the cells were treated with 50 μl of a 100 μg/ml sock of RNase. Then the cells stained with 200 μl PI (from 50 μg/ml stock solution). The samples were subsequently analyzed with a flow cytometer and data were analyzed using FlowJo software.

### Migration assays

24-Well transwells which contained a poly-carbonate membrane were used to test the migration of SMMC-7721 cells, as previously described [15]. 1 × 10^5^ cells cultured in 200 μl DMEM with 0.5% FBS were added to the upper compartment of the chamber, while the lower compartment was filled with 500 μl DMEM containing 5% FBS. After incubation at 37 °C for 24 h, the tumor cells remaining inside the upper chamber were removed with cotton swabs. The cells on the lower surface of the membrane were stained with 0.1% crystal violet after fixation with methanol, and then counted under a light microscope.

### Western blot analysis

The treated cells were lysed in an RIPA buffer and centrifuged at 12,000 rpm for 30 min. Supernatants were collected, and the total protein concentration was quantified using the bicinchoninic acid (BCA) assay kit. Equal amounts of proteins were then separated by SDS-PAGE gels and transferred to a PVDF membrane. After blocking with 5% skim milk at room temperature for 1–2 h, the membranes were incubated with primary antibodies against rabbit anti-active caspase-3, rabbit anti-caspase-9, rabbit anti-AKT, rabbit antiphospho-AKT and rabbit antiphospho-PI3 Kinase. (All of the above antibodies were procured from Cell Signaling Technology); equal lane loading was confirmed using a monoclonal antibody against β-actin (Proteintech). The membranes were then incubated in an HRP-conjugated anti-rabbit IgG for 1 h at room temperature. Chemiluminescence was detected using an ECL Western blotting substrate, and band intensity was assessed using a gel imaging analysis system (Syngene, UK). The relative expression of target protein was normalized to the expression of β-actin.

### Antitumor activity assessment of BA in vivo xenograft experiments

#### Animals

The tested female nude mice, aged 5 weeks and weighted 15–20 g, were provided by SLRC Laboratory Animal (China). The mice were allowed to acclimate to the environment in our animal facility for one week, after which procedures were performed according to the Guidelines for the Care and Use of Laboratory Animals published by the National Institutes of Health (NIH publication 86–23, revised 1986) and the animal regulations of Hunan Province, China. The mice were fed at the Animal Experiment Center of Central South University (Changsha, China), exposed to a 12-h environmental light cycle, housed at 22 ± 2 °C and 55 ± 5% humidity, and had free access to standard rat food and tap water in individual cages during the entire experimental period.

#### Tumor xenograft experiments

SMMC-7221 cells (0.2 ml, 1 × 10^8^ cells/mice) were injected subcutaneously into 20 nude mice until tumors reached the targeted volume of 100–150 mm^3^. The 20 mice were then randomly divided into four groups: DMSO/Olive oil (2%V/V) as the non-treated control group, 10 mg/kg 5-FU group, 5 mg/kg BA group and 10 mg/kg BA group. BA was dissolved in DMSO to a stock concentration of 100 mg/ml, and then was diluted with olive oil to the desired concentrations for intraperitoneal injection once a day for 5 consecutive days. The body weight as a indicators of BA toxicity was measured every 2 days for each mouse. Tumor volume in each mouse was measured and calculated every 2 days according to the formula previously described [16]: V = length × width^2^ × 1/2. After the animals were sacrificed, the tumors were collected, weighed and the tumor inhibitory rate was calculated by the formula: Tumor inhibitory rate % = 100 × (W_Control mice_ − W_Treated mice_)/W_Control mice_.

### Histopathology analysis

The liver, kidney, spleen and lung tissue from the mice were embedded in paraffinum and then sectioned at a thickness of 4 μm for pathological examination to evaluate the effect of BA on those organs. The sections were deparaffinized in xylene, rehydrated in ethanol, rinsed in distilled water, and then stained with hematoxylin and eosin (H&E), followed by dehydration in graded alcohol. Slides were mounted and analyzed under a microscope (Motic China Group Co., LTD, Fujian, China).

### Statistical analysis

All experiments were replicated at least 3 times, and the data was shown as mean ± standard deviation (SD). Statistical analysis was performed using the SPSS16.0 software (SPSSInc., Chicago, USA). Differences among the samples were evaluated by one-way analysis of variance (ANOVA). A statistically significant difference was assumed at p < 0.05.

## Results

### BA inhibited the proliferation of both cancerous and normal liver cells

**Fig. 1:**
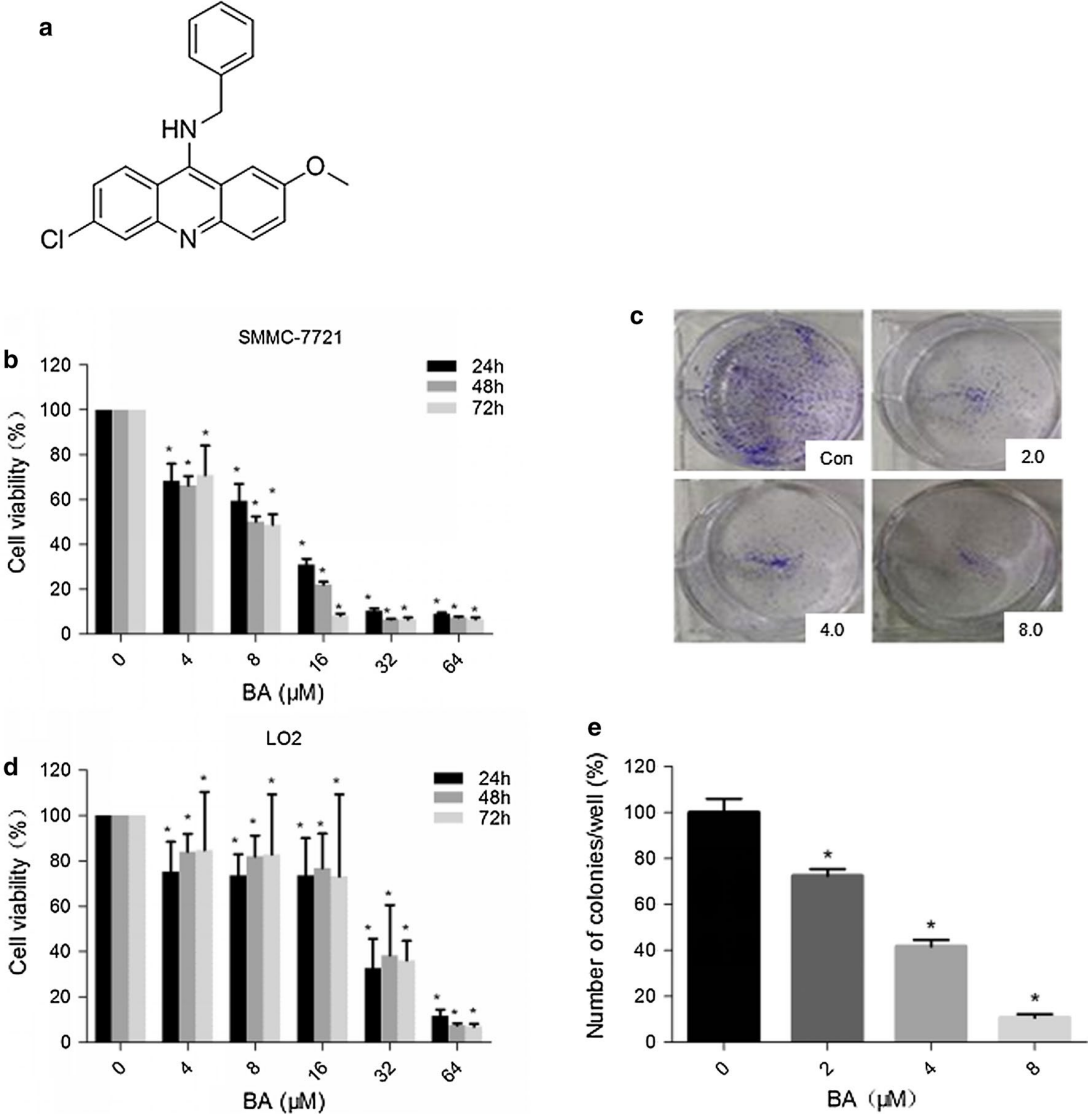
The effect of BA on cell viability. **a** Structure of BA. Chemical formula: C_21_H_17_ClN_2_O; molecular weight: 348.5. **b**, **d** SMMC-7721 and LO2 cell viability at 24th, 48th, and 72th hour after BA treatment. SMMC-7721 and L02 cells were cultured in 96-well plates in the presence of a series of BA concentrations: 0, 4, 8, 16, 32 and 64 μM. Cell viability was measured by MTT assay. The data was obtained from three independent experiments and was presented as mean ± SD (*p < 0.05, BA vs. control). **c**, **e** Colony formation of SMMC-7721 cells in BA treatment. BA concentrations were 2, 4, and 8 μM. No BA treatment cells were used as negative control. SMMC-7721 cells (1*10^3^ cells/well) were seeded and cultured in the absence or presence of BA at increasing concentrations (0, 2, 4, and 8 μM) for 24 h. Colonies were stained with* crystal violet* and quantified by densitometry (*p < 0.05, BA vs. control). **c** Representative pictures of colonies formed. **e** Percentage of colonies formed per well

To examine the inhibitory effect of BA on the proliferation of cancerous and normal hepatocytes, we treated HCC derived SMMC-7721 cells and normal hepatocyte LO2 cells with increasing doses of BA for 24, 48, and 72 h. As a result, SMMC-7721 cells and LO2 cells both showed reduced cell viability with the increase of BA concentrations. However, SMMC-7721 cells were more sensitive to BA compared with LO2 cells at BA concentrations between 4 and 32 μM (Fig. 1b, d). These results indicated that BA was less toxic to normal cells compared to the tumor cells.

### BA inhibited the colony formation of SMMC-7721 cells

In order to investigate the anti-cancer function of BA, we first performed colony formation assays on SMMC-7721 hepatocarcinoma cells. Our results showed that BA inhibited the colony formation rate of SMMC-7721 cells in a dose dependent manner. The higher the BA doses used, the fewer colonies SMMC-7221 cells formed (Fig. 1c, e).

### BA inhibited the migration of SMMC-7721 cells

**Fig. 2:**
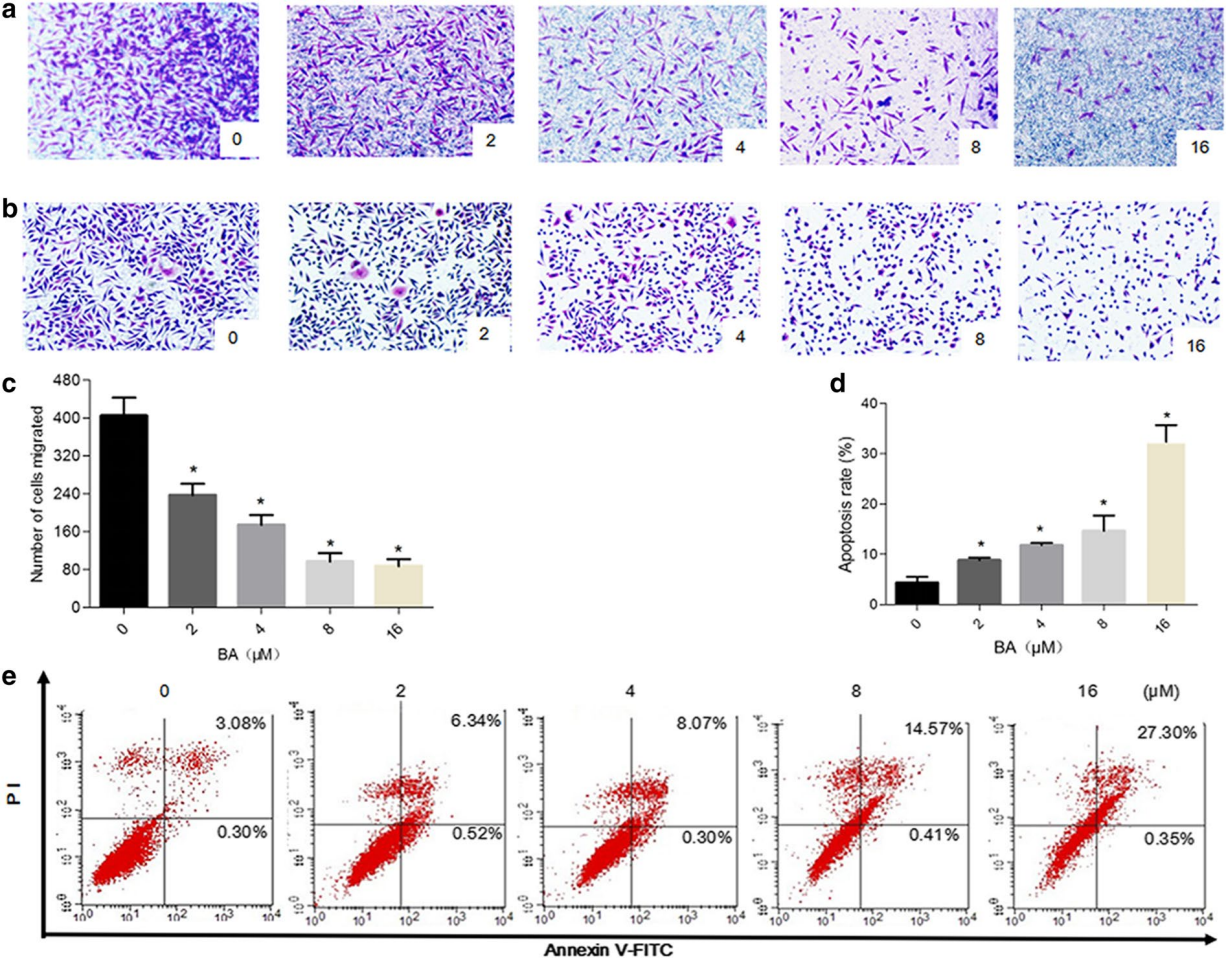
Effect of BA on SMMC-7721 cell migration and apoptosis. **a**, **b** SMMC-7721 cells treated with various concentrations of BA (0, 2, 4 and 8 μM) for 24 h. **a** Representative images of SMMC-7221 cells number in the transwell chamber membrane after BA treated. **b** Hematoxylin & eosin staining showing the morphological changes of SMMC-7721 cells after BA treatment. **c** Quantification of the migration of SMMC-7721 cells; data here represents replicates of three independent experiments and is expressed as the means ± standard deviation. **d** Apoptosis rate of SMMC-7221 cells induced by increasing concentrations of BA. Data here represents replicates of three independent experiments, and is expressed as the mean ± standard deviation. Data was analyzed using the Student’s t test. **e** Annexin V-FITC/Propidium iodide (PI) staining showing the apoptosis rate of SMMC-7721 cells after BA treatment (0, 2, 4, 8, 16 μM) for 24 h. *p < 0.05, compared to the control group

Next, we studied the function of BA to reduce SMMC-7721 cell migration using the Boyden chamber transwell. Our data showed that BA was able to inhibit cell migration in a dose dependent manner:the higher of BA concentrations, the smaller number of cells that migrated (Fig. 2a, c) (number of cell migration to transwell chamber, 0 vs. 2 vs. 4 vs. 8 μM: 405 ± 38 vs. 237 ± 24 vs. 174 ± 21 vs. 97 ± 18, p < 0.05).

### BA induced SMMC-7721 cell apoptosis and resulted in cell cycle arrest in G1 phase

Then We investigated whether BA applies its anti-tumor function by inducing apoptosis. In order to study this, we measured the apoptosis rate of SMMC-7721 cells after treating them with increasing concentrations of BA for 24 h. Morphological changes of these cells were observed by Hematoxylin and eosin staining and the apoptosis rate was measured by an Annexin V-FITC/PI staining assay. With increasing concentrations of BA, more cells showed volume shrinkage, chromatin condensation, and cell debris (Fig. 2b). Our results also showed that BA induced apoptosis in SMMC-7721 cells, and the apoptosis rate increased with increasing concentrations of BA [0 vs. 2 vs. 4 vs. 8 vs. 16 μM: (4.31 ± 1.20) % vs. (8.80 ± 0.54) % vs. (11.82 ± 0.47) % vs. (14.64 ± 3.07) % vs. (32.39 ± 12.84) %, p < 0.05] (Fig. 2d, e).

**Fig. 3:**
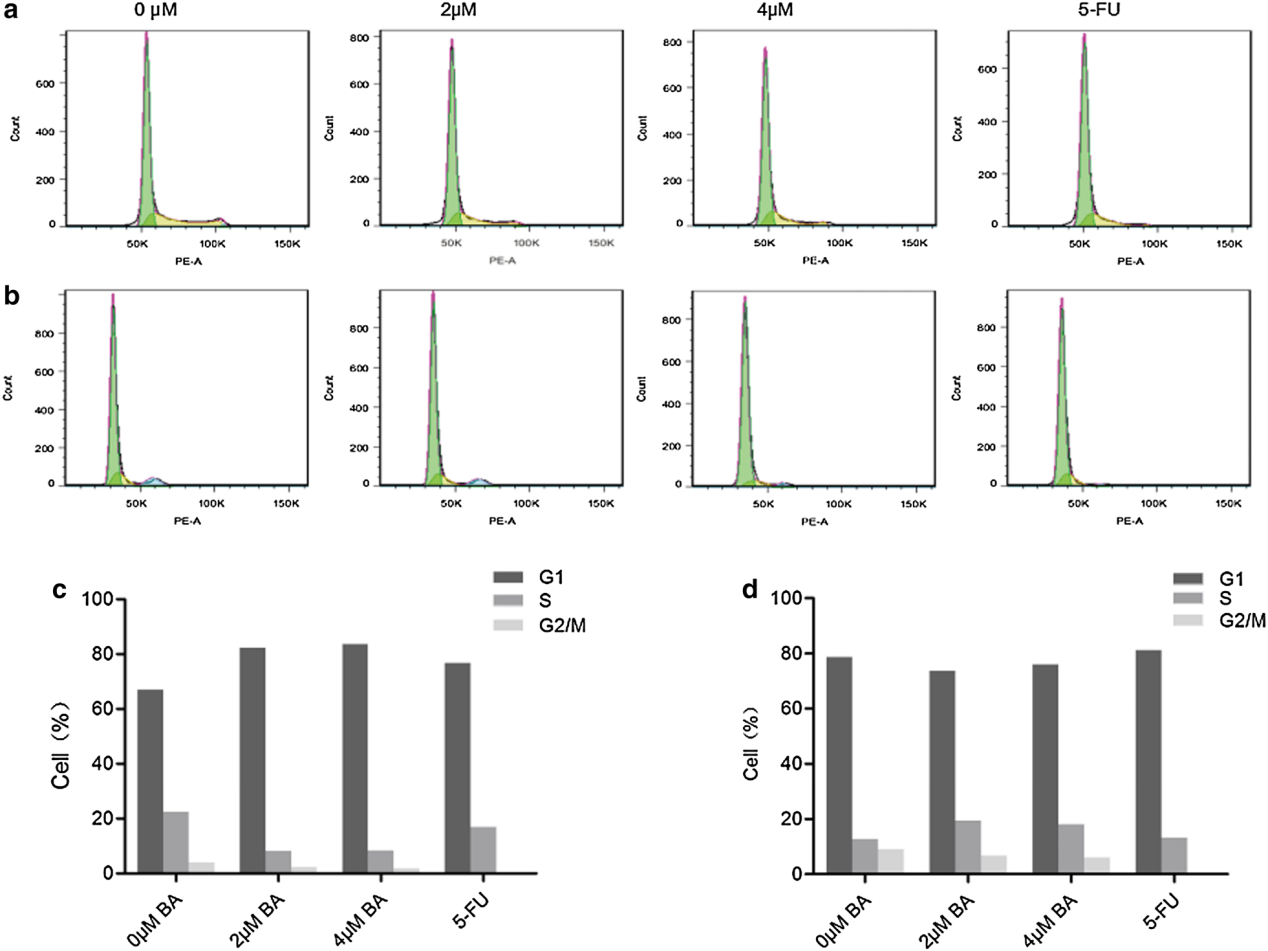
Effect of BA on cell cycle arrest. **a**, **b** The percentage of cells in G1, S, or G2/M phase in SMMC-7721 or LO2 cells treated with various concentrations of BA (0, 2 and 4 μM) and 5-FU (10 μM) for 24 h as detected by flow cytometry. **c**, **d** Histograms showing the percentage of cells in G1, S, and G2 phases

To study the antiproliferative mechanism of BA in SMMC-7721 cells, we tested whether BA treatment affects the SMMC-7721 cells cycle. Cells were cultured with 0, 2, 4 μM BA or 5 μM 5-FU for 24 h, and cell cycle arrest was analyzed with flow cytometry. As shown in Fig. 3, BA treatment resulted in cell cycle arrest in G1 phase compared with control. The percentage of cells in G1 in 0 vs. 2, 4 μM BA and 5-FU groups was 66.0% vs. 82.2% vs. 83.6% and 76.7% (Fig. 3)

### BA induced apoptosis is caspase dependent and involves the PI3K/AKT pathway

**Fig. 4:**
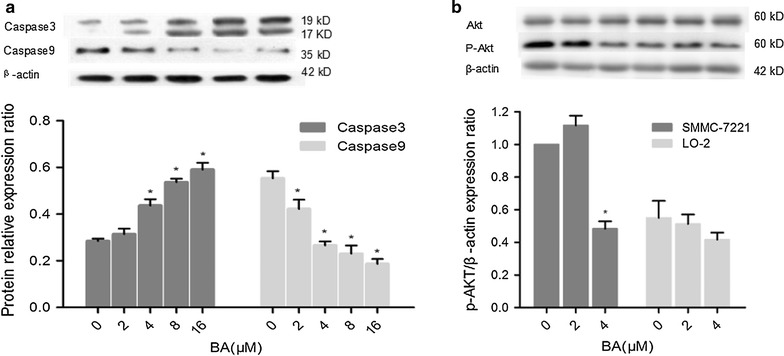
Mechanistic studies of BA anti-hepatocellular carcinoma effect. **a** BA induced caspase 3 expression but reduced the caspase 9 level in SMMC-7721 cells. SMMC-7721 cells were treated with increasing concentrations of BA (0, 2, 4, 8, 16 μM) for 24 h before analysis; β-actin was used as a loading control. **b** P-Akt and Akt expression in SMMC-7721 and LO2 cells treated with increasing concentrations of BA (0, 2, 4 μM) for 24 h

After determining that BA induced apoptosis in SMMC-7721 cells, we next investigated whether caspase family proteins were involved in the apoptosis. We treated SMMC-7721 cells with increasing concentrations of BA for 24 h, and then measured the expression of caspase 3 and caspase 9. Compared to the non treated control, SMMC-7721 cells treated with BA at concentrations of 4, 8 and 16 μM showed significantly increased caspase 3 expression, but significantly decreased caspase 9 expression (p < 0.05) (Fig. 4a) This result indicated that BA-induced apoptosis was caspase protein dependent.

**Fig. 5:**
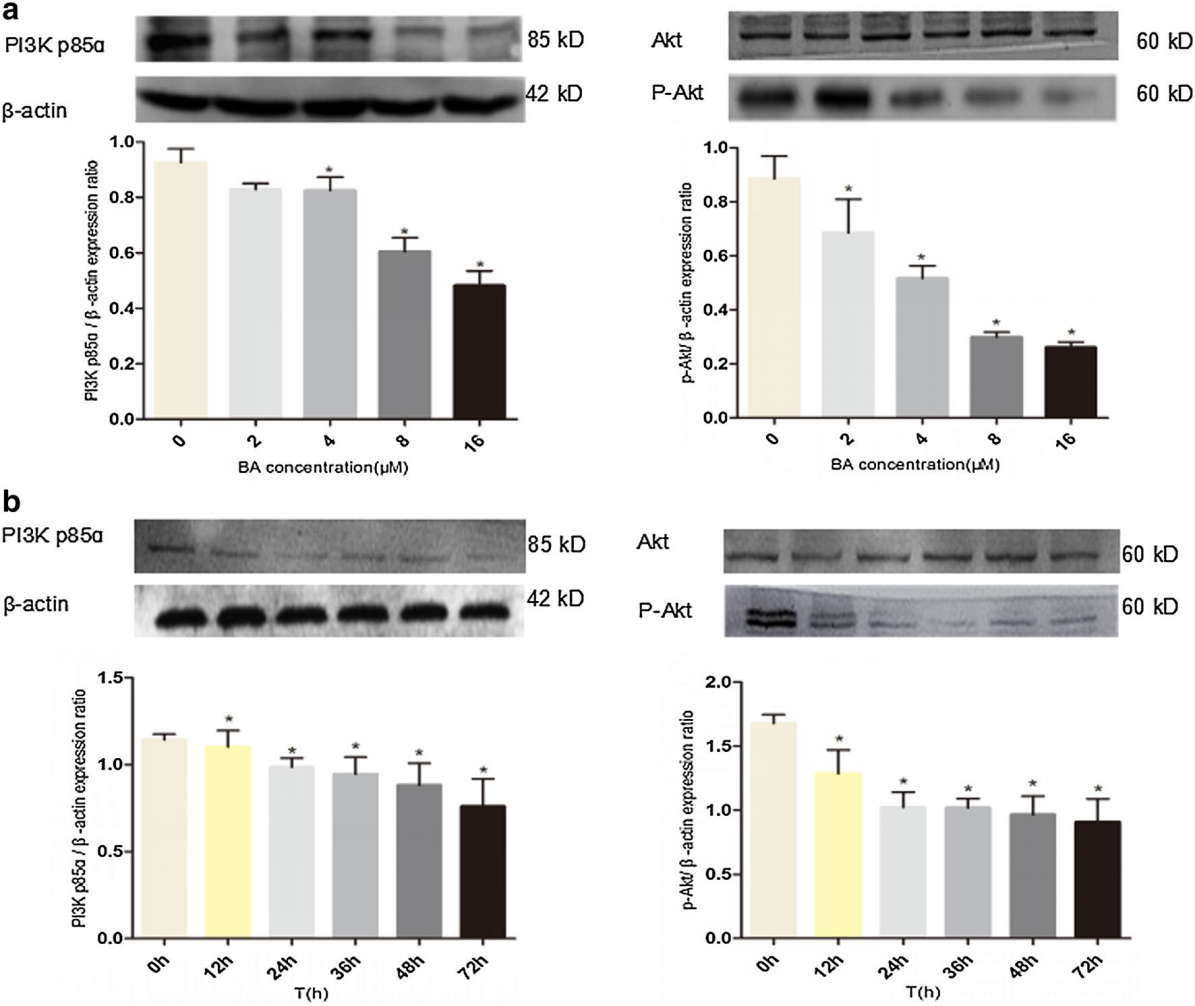
Mechanistic studies of BA anti-hepatocellular carcinoma effect. **a** PI3K p85ɑ, P-Akt and Akt expression in SMMC-7721 cells treated with increasing concentrations of BA (0, 2, 4, 8, 16 μM) for 24 h. **b** The change of PI3K p85α, P-Akt and Akt expression in BA treated SMMC-7721 cells over time. SMMC-7721 cells were treated with 8 μM BA, and their PI3K p85α, P-Akt and Akt expression were measured at 0, 12, 24, 36, 48, 72 h after the treatment. β-actin was used as a loading control

Since the PI3K/Akt pathway plays a critical role in regulating the survival/death of HCC cells [17], we investigated whether BA employed its cytostatic and cytotoxic functions through Akt-dependent signaling. Our precious data had showed that the lower cytotoxic action of BA in LO2 cells, and the results of the effect of cells cycle showed that BA had little effect on LO2 cells cycle compared with SMMC-7221 cells (Figs. 1b, d, 3d). Besides, the results of WB showed that phosphorylated Akt (p-Akt) was around 2 times higher in cancer SMMC-7721 cells than non-tumour LO2 cells. And p-Akt was significantly reduced in SMMC-7721 cells by treated 4 μM BA, while p-Akt in LO2 cells was little changed by treated different concentration BA (Fig. 4b). In addition, our results also revealed that the p-Akt and PI3K p85α were markedly decreased in SMMC-7721 cells treated with increasing concentrations and the time of BA treated. However, total Akt levels in SMMC-7721 cells remained unchanged in response to BA (Fig. 5a, b). Above those results, we thought that the BA cytotoxicity perhaps correlated with lower effect on p-Akt levels.

### BA suppressed tumor growth in vivo

**Fig. 6:**
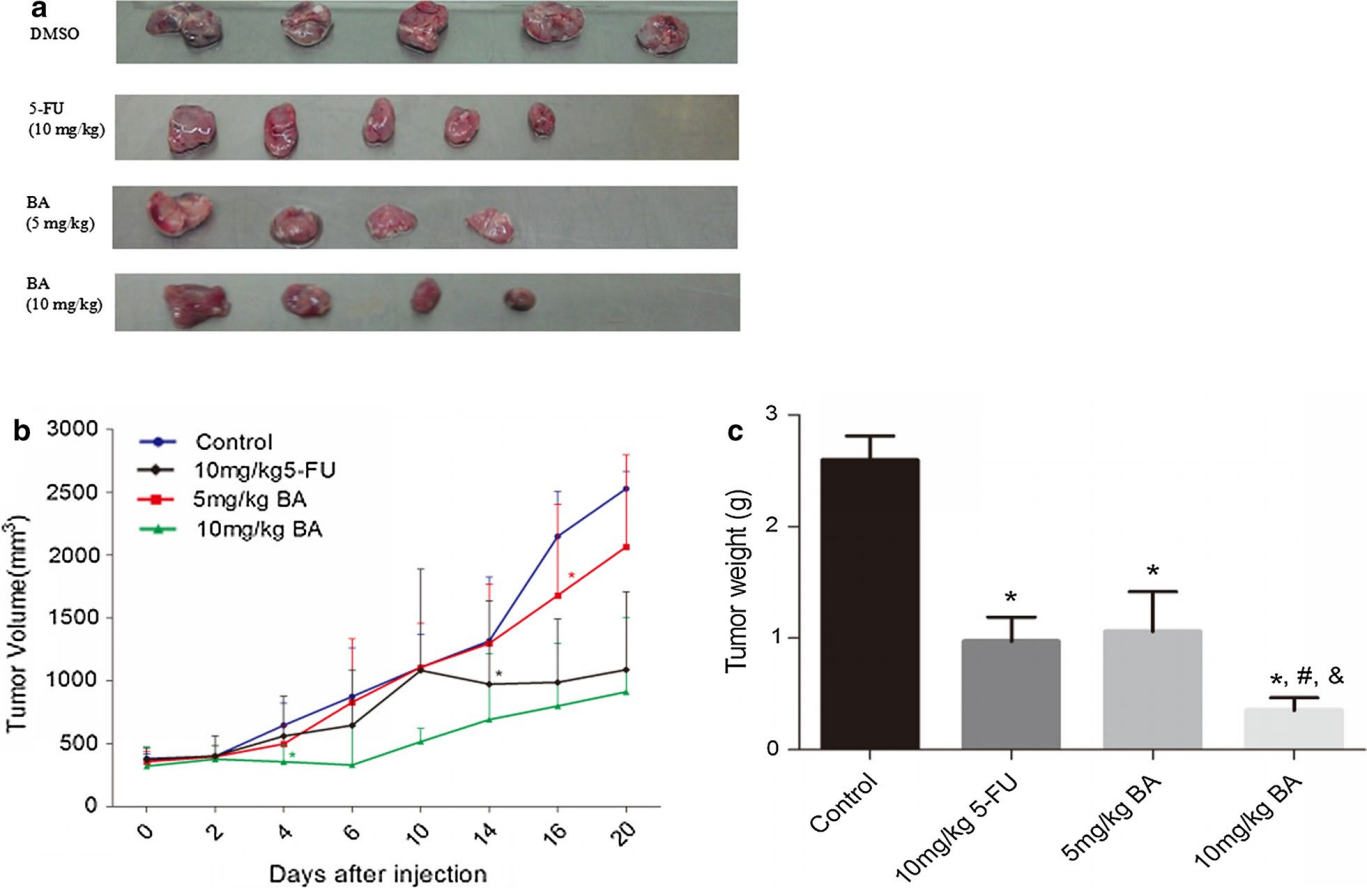
BA significantly inhibited the growth of hepatocellular carcinoma xenografts in vivo. Nude mice bearing hepatocellular carcinoma tumor xenografts were treated with DMSO/Olive oil (2%V/V, as the non-treated control), 5-FU (10 mg/kg i.p.) or BA (5, 10 mg/kg i.p.) once a day for 5 days. Tumor volumes and weights were measured every 2 days. **a** Representative images of tumors treated with 5-FU (10 mg/kg) and BA (5, 10 mg/kg) on day 20, DMSO treated tumors were included at negative control. **b** Tumor volume over time. *Asterisk* from the first day the tumor volume was significantly different compared with the control group. **c** Tumor weight at the end of the experiment. Tumor volumes and weights here are presented as mean ± SD; *p < 0.05, vs. control, ^#^ p < 0.05, vs. 10 mg/kg 5-FU, ^&^ p < 0.05, vs. 5 mg/kg BA

We determined that BA suppressed the growth of cancerous liver cells by inducing apoptosis and cell death. Next, we investigated whether BA showed the same inhibitory function on tumor growth in vivo. In order to study this, nude mice were preconditioned by subcutaneous injection of SMMC-7221 cells (0.2 ml, 1 × 10^8^ cells/mice) until tumor size reached the targeted volume of 100–150 mm^3^. Then, two concentrations of BA (5 and 10 mg/kg) or 5-FU (10 mg/kg) were injected into the mice intraperitoneally once a day respectively for 5 days. The growth of tumors in mice treated by 5-FU (10 mg/kg), BA (5 mg/kg) or BA (10 mg/kg) was largely suppressed from the fourteenth, sixteenth and twentieth day, respectively, while the tumor size of the control mice gradually increased over time (p < 0.05). On day 20, the mean tumor volumes treated with 10 mg/kg 5-FU reached a size of 1087.18 mm^3^, those treated with 5 mg/kg BA reached 2048.20 mm^3^, and those treated with 10 mg/kg BA reached 912.28 mm^3^. The tumor size of all the treated mice was much less than that of the control group, which reached a volume of 2526.176 mm^3^. The differences in tumor volume among the control group, BA treated groups and 5-FU treated group can be significantly visualized (p < 0.05, Fig. 6a). Furthermore, the volumes of 10 mg/kg BA treated tumors were less than those of the 5-FU treated tumors (p < 0.05, Fig. 6b), which indicated a stronger inhibitory function of BA compared to the traditional chemotherapeutic drug.

At the end of our experiments, the tumors were excised from each sacrificed mouse and weighed. Results showed that BA at concentrations of 5, 10 mg/kg and 5-FU at a concentration of 10 mg/kg significantly inhibited the growth of liver tumors (p < 0.05) (Fig. 6c). The mean tumor weight of the control group was 2.59 ± 0.49 g, in comparison to 1.06 ± 0.71, 0.61 ± 0.11 and 0.97 ± 0.49 g for tumors in the BA (5 and 10 mg/kg) treated group and the 5-FU (10 mg/kg) treated group, achieving a tumor inhibitory rate of 58.24 ± 27.51, 86.49 ± 9.63 and 63.42 ± 19.24% for each group, respectively (p < 0.05). Furthermore, our results showed that 10 mg/kg BA administration is more efficient than 10 mg/kg 5-FU in inhibiting tumor growth (p < 0.05).

### The toxicity of compound BA on mice

**Fig. 7:**
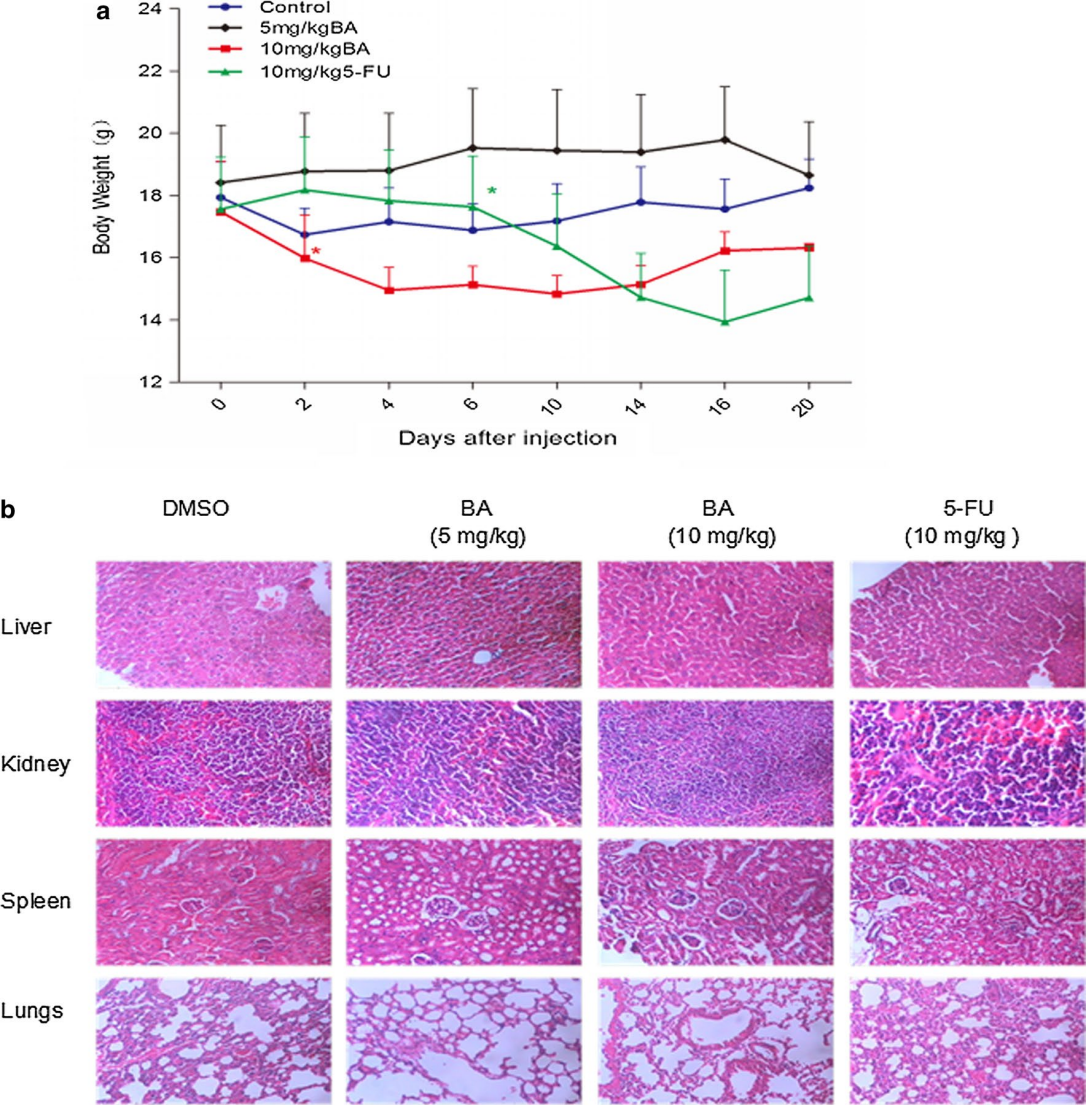
The effect of BA on body weight, liver, kidney, spleen and lung functions of mice. Nude mice bearing hepatocellular carcinoma tumor xenografts were treated with DMSO/normal saline (as the non-treated control), 5-FU (10 mg/kg) or BA (5, 10 mg/kg) separately once a day for 5 days. Their body weight, liver, kidney, spleen and lung functions were measured at the day 20. **a** Changes in body weight of nude mice. *Asterisk* from the first day the body weight of mice was significantly different compared with the control group. **b** The hematoxylin and eosin staining of the liver, kidney, spleen and lungs. *p < 0.05, vs control

To monitor the toxicity of BA, the body weight of each mouse was measured. The average body weight of mice treated with 10 mg/kg BA was significantly less than that of the control group (Fig. 7a), but it was less compared with the body weight changes in the 5-FU group during the experiment.

As shown in Fig. 7b, after hematoxylin and eosin staining of the liver, kidney, spleen and lungs of the mice, there were no significant pathological changes observed compared with the control group. Furthermore, the examination results from Table 1 showed that BA had no effect on the liver and kidney function of treated tumor mice. So no evident toxicity was identified in the treated animals of BA compared to the control or 5-FU treated group.

**Table 1:** The effect of BA on the liver and kidney function of mice

Group	ALT (U/L)	A/G	TBIL (μmol/L)	AST (U/L)	BUN (mmol/L)	CR (μmol/L)
Control	71.0 ± 18.2	1.70 ± 0.23	1.94 ± 0.67	266 ± 70.11	8.43 ± 1.20	13.60 ± 6.47
BA (5 mg/kg)	73.8 ± 39.4	1.46 ± 0.25	0.94 ± 1.61	174 ± 118	8.67 ± 3.77	4.00 ± 2.00
BA (10 mg/kg)	92.2 ± 13.6	1.65 ± 0.17	1.70 ± 0.97	356 ± 92.6	9.85 ± 1.86	13.40 ± 7.02
5-Fu (10 mg/kg)	98.0 ± 34.7	1.40 ± 0.10	0.20 ± 0.00*	549 ± 361	15.1 ± 8.06	8.33 ± 5.77

*ALT* alanine aminotransferase, *A/G* the proportion of albumin and globulins, *TBIL* total bilirubin, *AST* aspartate aminotransferase, *BUN* blood urea nitrogen, *CR* creatinine

* *p* < 0.05, compared to control group

## Discussion

BA is a novel compound discovered in the sessile organism sponges. Its function in anti-angiogenesis has been reported in Human Umbilical Vein Endothelial Cells (HUVECs) [18]. Since angiogenesis is involved in many types of cancer, it is reasonable to assume that BA might be able to treat cancer. Therefore, in our study, we examined BA’s anti-cancer role in HCC both in vitro and in vivo.

According to our in vitro study, BA significantly induces apoptosis and migration of SMMC-7721 cells, and therefore, inhibits their proliferation and survival. This inhibitory effect is dose dependent. Mechanistic studies using western blot analysis revealed that this pro-apoptotic effect occurs correlated with the PI3K-AKT pathway, where BA remarkably decreases the level of caspase-9 and activates caspase-3 expression in SMMC-7721 cells. In vivo, the growth of xenograft tumors was remarkably inhibited by intraperitoneal injection of BA. We just simply assume that weight change of mice is an indicator of the drug toxicity for their living and genetic environment are similar. So the effect of inhibition measured by tumor volumes and weights and study of mice weights revealed that BA (10 mg/kg) might have a higher efficacy than 5-FU (10 mg/kg), although it may cause higher toxicity at the beginning of treatment. However, the toxicity reduced over several days, possibly due to adaptation. On the contrary, the toxicity of 5-FU increased during the treatment, and surpassed that of BA starting at the 14th day (Fig. 4b). Of course the toxicity of BA need more trials to study, ours is just a simple exploratory trial here.

The different efficacy of BA and 5-FU may be related to their unique mechanisms. 5-FU is a popular broad-spectrum chemotherapy drug for various types of cancer. It is a thymidylate synthase inhibitor, which blocks the synthesis of pyrimidine and thymidine, a nucleoside for DNA replication, and therefore leads to cancer cell death. Meanwhile, 5-FU also causes extensive side effects for off site targets and destroys rapidly dividing normal cells in the patients aside from tumors and results in proliferative inhibition, DNA damage and cell death [19]. On the contrary, BA may have an anti-HCC effect via the PI3K-AKT pathway according to our data. Recent research revealed that the PI3K-AKT signaling pathway is inappropriately activated in many types of cancer [20]. Chen et al. claimed that p-AKT expression had a positive association with tumor grade, presence of intrahepatic metastasis and vascular invasion [21]. Indeed, the molecular mechanics of the PI3K/AKT pathway have been shown to promote cell proliferation and survival [21]. Thus, BA may be an optimal therapeutic drug for targeting this pathway for HCC.

In addition, studies have indicated that the PI3K/Akt signaling network is aberrantly up-regulated in the neoplasia of HCC [22]. The PI3K/Akt signaling pathway also plays a key role in cancer cells’ survival in response to DNA damage by controlling FANCD2 and ribonucleotide reductase (RNR). FANCD2 plays an important role in the activation of DNA damage checkpoints, and RNR is critical for the completion of DNA replication and repair in response to DNA damage and replication stress [23]. Meanwhile, 5-FU mainly affects DNA synthesis of cell replication. Furthermore, the PI3K-Akt pathway also promotes cell cycle progression by affecting cyclin D1, which induces a G1-phase arrest and results in a reduction of the S-phase subpopulation [23], while 5-FU has an opposite effect [24]. Therefore, the drugs targeting PI3K/AKT/mTOR may be more effective than broad-spectrum chemotherapy such as 5-FU in prevention of liver cancer.

In recent years, blockade of PI3K/AKT/mTOR signaling appears to be an attractive therapeutic strategy in HCC [25]. Several inhibitors targeting this pathway have been recently discovered, some of which are being evaluated in clinical trials. Perifosine, a synthetic alkylphosphocholine anti-tumor agent, has been reported as a promising drug for treatment of HCC characterized in Phase II clinical trials [26, 27]. Furthermore, MK-2206, an allosteric Akt inhibitor, could be a valuable compound for treating HCC patients displaying down-regulation of the phosphorylation levels of Akt-1 synergized and is currently being used in preclinical settings [28]. Since BA can inhibit Akt phosphorylation, it is an attractive therapeutic treatment for HCC.

In our study, the physiological function of liver, kidney and lung tissue examined by H&E staining showed no obvious differences among BA (5 and 10 mg/kg), 5-FU (10 mg/kg) and the non-treated control. However, previous research has shown that 5-FU causes nephrotoxicity [19] and hepatotoxicity [29]. These contradictory results may be due to the higher concentration of 5-FU used in these studies. 150 mg/kg 5-FU was used to study induced renal toxicity and liver damage [19, 29], compared to our concentration of 10 mg/kg. When we compare studies that used the same 5-FU concentration as we did, we draw the same conclusion. Mandziuk et al. [30] showed that animals treated with 5-FU (10 mg/kg) did not show any hepatic function disorders, and found no incidence of histological abnormalities such as necrosis or fibrosis.

Recently, sponge derived products have become an important source for anticancer drug discovery due to their novel structure and various biological properties. Besides our compound, BA, that exhibits strong anti-HCC activity, Eribulin mesylate (E7389), a nontaxane microtubule dynamic inhibitor extracted from the marine sponge *Halichondria okadai*, increased the survival rate for metastatic breast cancer patients in Phase I–II clinical trials [31]. Zalypsis, a synthesized alkaloid derived from sponge extracted compounds showed potent anti-myeloma activity [32].

As an exploratory study, our results showed that BA could be a promising candidate for an anti-HCC drug by targeting the PI3K/Akt pathway. In order to have a comprehensive evaluation of BA, further investigation is needed in the following areas. First, to determine whether BA also affects other cancerous functions of SMMC-7221 cells and HUVECs such as angiogenesis [18], Second, to determine whether BA targets other signaling pathways to inhibit HCC development. Third, the safety of BA as an anti-cancer drug needs more evaluation, preferably in animal studies. In our study, we used a BA concentration of up to 10 μg/ml, and H&E staining on liver, kidney and lung tissue did not show any damage. However, it is highly possible that a higher BA concentration is necessary in eliminating in vivo tumors. Moreover, other tests besides H&E staining are also worth considering. Furthermore, the adaptation of BA toxicity during mice study needs further investigation. BA exhibits higher toxicity than 5-FU in the beginning of treatment, but its toxicity decreases over time. Longer BA treatment might be necessary to test the correlation between BA toxicity and efficacy. Finally, other widely used cancer drugs need to be compared with BA to evaluate the efficacy and toxicity of BA.

## Conclusions

Our study demonstrated that BA inhibits Akt phosphorylation and exerts potent anticancer functions in HCC by down-regulating the PI3K pathway, which results in inhibited cell proliferation and increased apoptosis in HCC cells and xenograft mouse tumors. These findings suggest that BA is a novel anticancer agent with potential clinical applications in treating HCC and other indications featured with down-regulated PI3K signaling.

## Additional files

**Additional file 1: M1.** Structure identification of compounds BA by mass spectrum. BA(C21H17ClN2O) m/z: calculated for [M+H]+: 349.5, found [M+H]+: 349.2.

**Additional file 2: M2.** Structure identification of compounds BA by Hydrogen spectrum. ^1^H NMR(500 MHz, TMS): δ 8.40(d, 1H), 7.90(d, 2H), 7.70(d, 1H), 7.65(s, 1H), 7.55(d,2H), 7.45(d,2H), 7.30(d,2H), 5.0(s,3H), 4.30(s,1H), 3.40(s, 2H).

**Additional file 3: M3.** Structure identification of compounds BA by Carbon spectrum. ^13^C NMR(125 MHz, TMS): δ 174.14, 167.47, 155.27, 150.52, 140.56, 134.04, 131.96, 128.97, 127.38, 126.50, 124.75, 123.34, 117.29, 115.06, 101.29, 61.79, 55.75, 49.01, 30.48, 29.32, 17.59.

## Abbreviations

BA: 6-chloro-2-methoxy-*N*-(phenylmethyl)-9-acridinamine
BCA: bicinchoninic acid
FITC: fluorescein isothiocyanate
HCC: human hepatocellular carcinoma
HUVECs: human umbilical vein endothelial cells
H&E: hematoxylin and eosin
MS: mass spectrometry
NMR: H-nuclear magnetic resonance spectroscopy
PI: propidium iodide
RNR: ribonucleotide reductase
