# Supplementary figures and images for: Induction of apoptosis and proliferation inhibition of hepatocellular carcinoma by 6-chloro-2-methoxy-N-(phenylmethyl)-9-acridinamine (BA): in vitro and vivo studies
Source: Cancer Cell Int. 2017 Jul 3;17:66. doi: 10.1186/s12935-017-0435-5 (PMC5496258; doi:10.1186/s12935-017-0435-5)

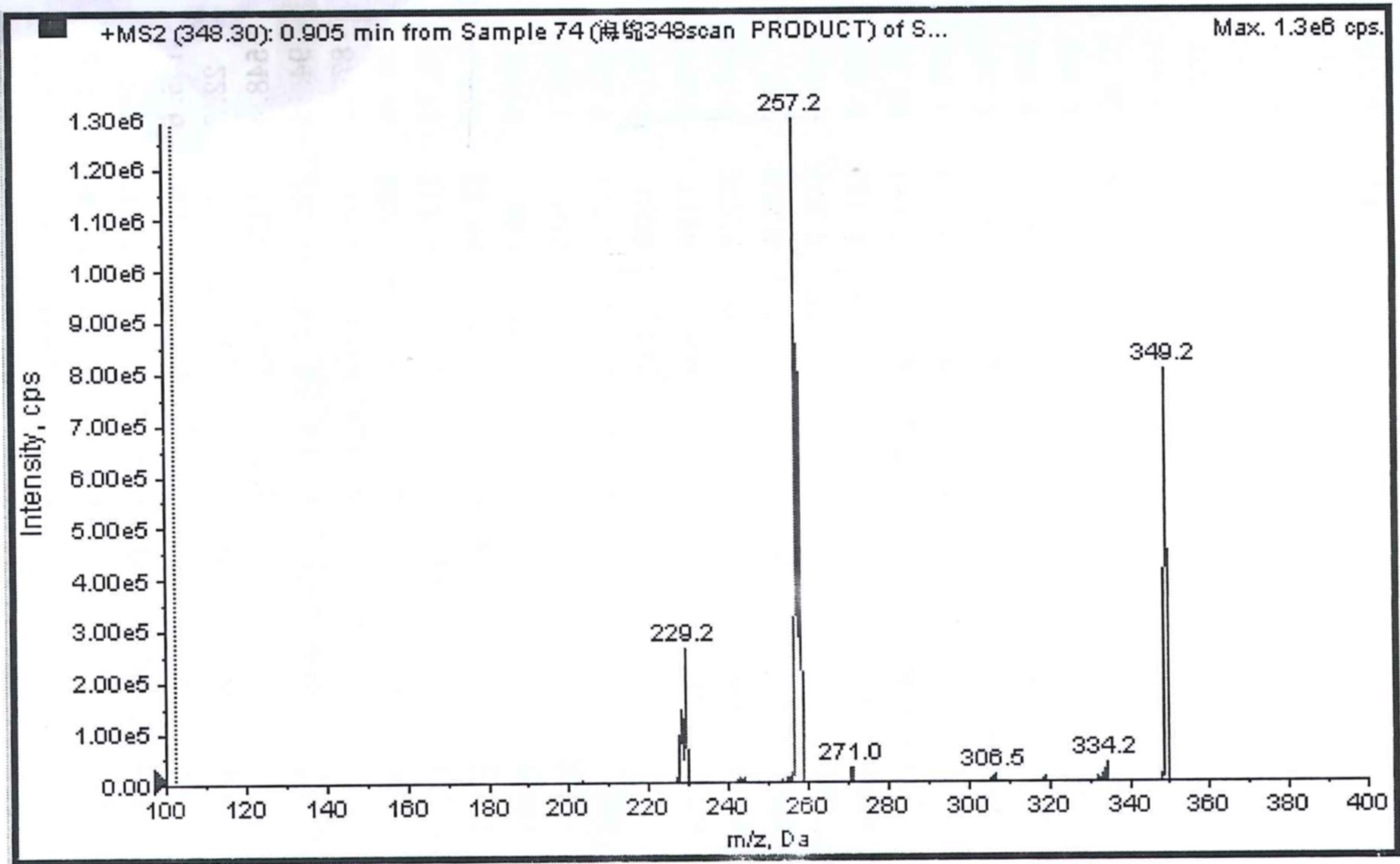

Supplement: Supplementary file 1 — Additional file 1: M1. Structure identification of compounds BA by mass spectrum. BA(C21H17ClN2O) m/z: calculated for [M+H]+: 349.5, found [M+H]+: 349.2. [file 12935_2017_435_MOESM1_ESM.tif]

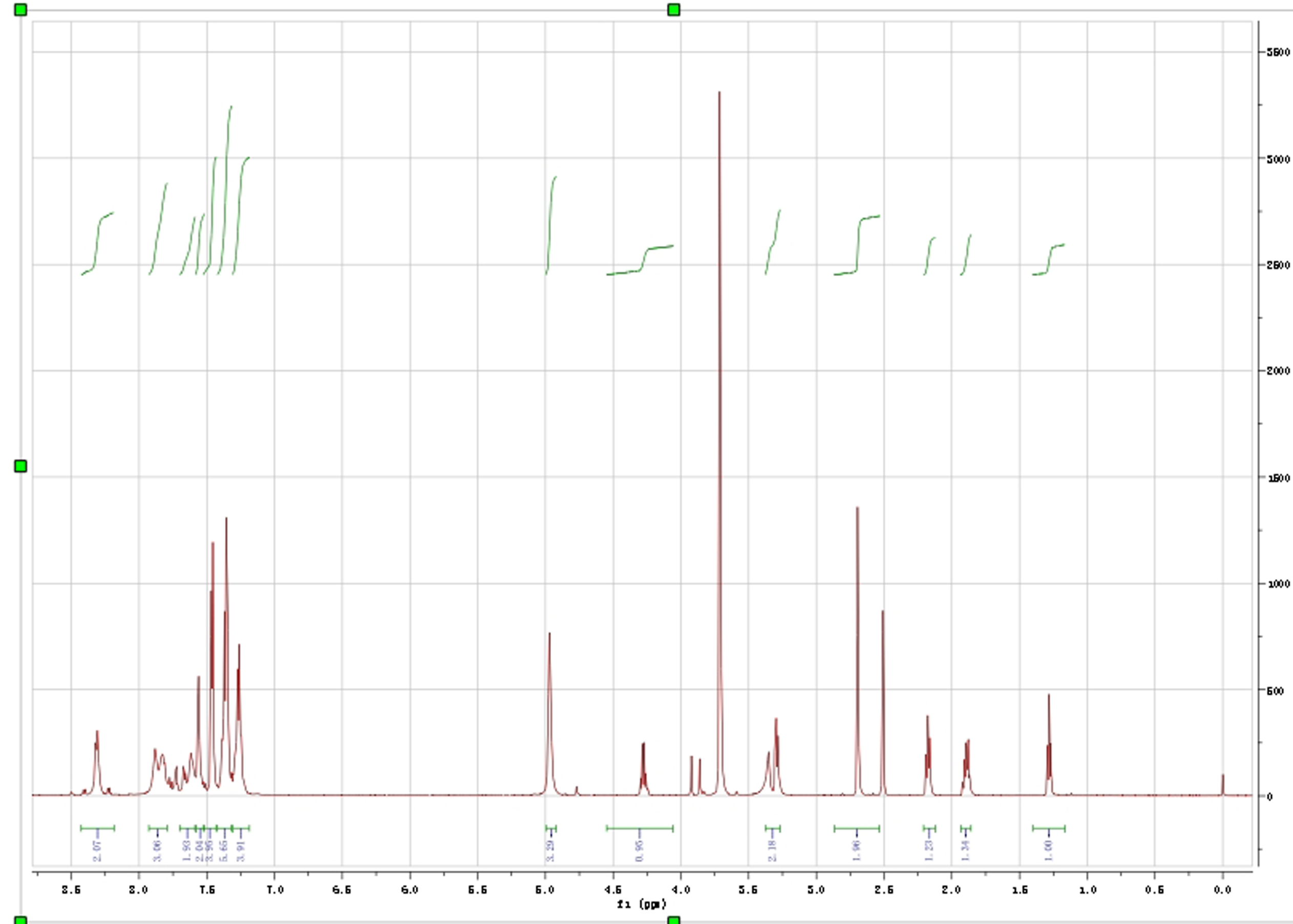

Supplement: Supplementary file 2 — Additional file 2: M2. Structure identification of compounds BA by Hydrogen spectrum. 1H NMR(500 MHz, TMS): δ 8.40(d, 1H), 7.90(d, 2H), 7.70(d, 1H), 7.65(s, 1H), 7.55(d,2H), 7.45(d,2H), 7.30(d,2H), 5.0(s,3H), 4.30(s,1H), 3.40(s, 2H). [file 12935_2017_435_MOESM2_ESM.tif]

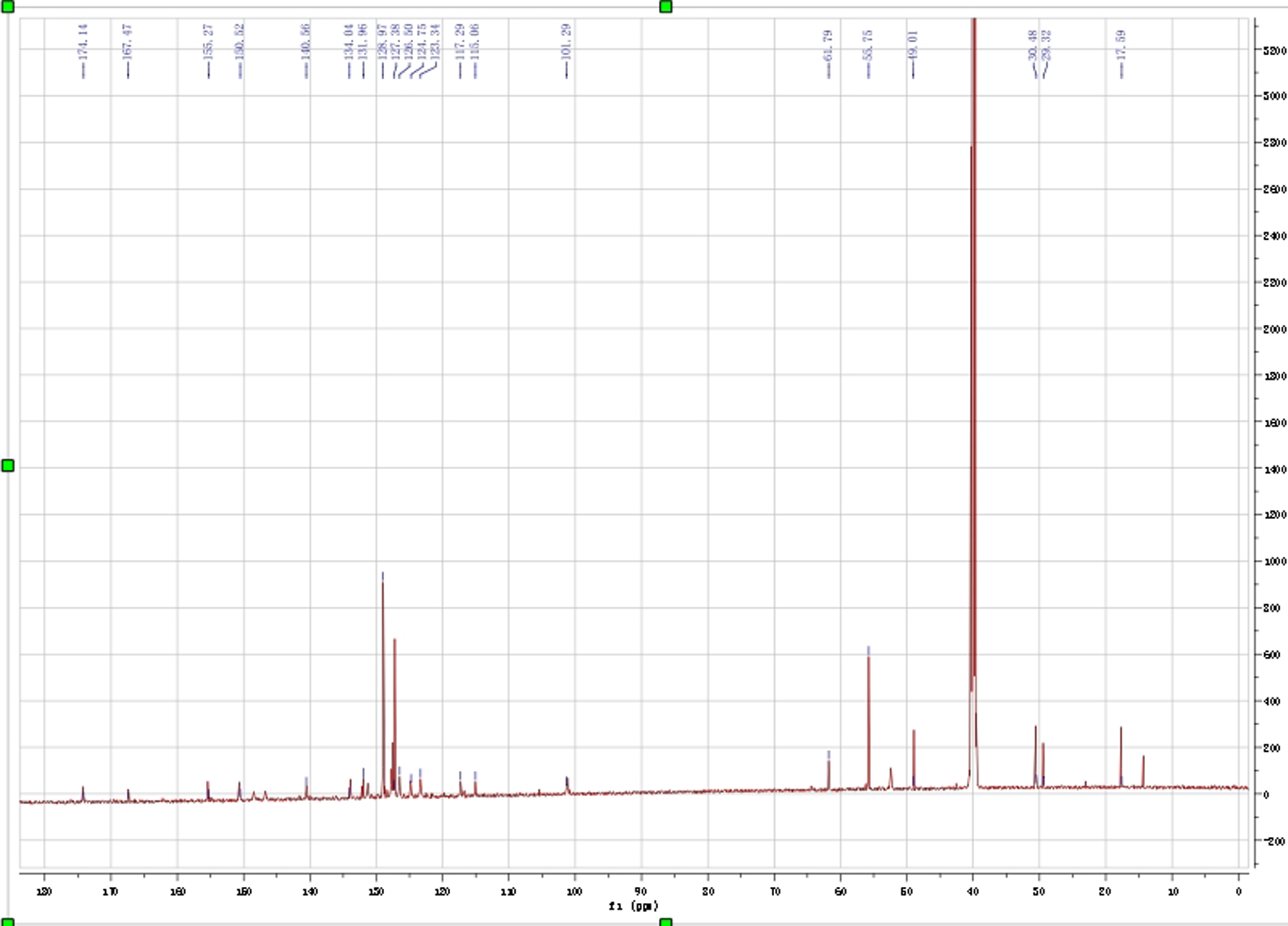

Supplement: Supplementary file 3 — Additional file 3: M3. Structure identification of compounds BA by Carbon spectrum. 13C NMR(125 MHz, TMS): δ 174.14, 167.47, 155.27, 150.52, 140.56, 134.04, 131.96, 128.97, 127.38, 126.50, 124.75, 123.34, 117.29, 115.06, 101.29, 61.79, 55.75, 49.01, 30.48, 29.32, 17.59. [file 12935_2017_435_MOESM3_ESM.tif]
